# Antioxidant and reduced skin-ageing effects of a polyphenol-enriched dietary supplement in response to air pollution: a randomized, double-blind, placebo-controlled study

**DOI:** 10.29219/fnr.v65.5619

**Published:** 2021-03-29

**Authors:** Vincenzo Nobile, Irene Schiano, Ana Peral, Silvana Giardina, Eleonora Spartà, Nuria Caturla

**Affiliations:** 1 Monteleoder S.L., Elche, Alicante, Spain; 2 Complife Italia Srl, San Martino Siccomario, Pavia, Italy

**Keywords:** air pollution, skin, nutraceutical, plant polyphenols, antioxidant, anti-ageing, clinical trial

## Abstract

**Background:**

Air pollution exposure is one of the major threats to skin health and accelerates skin ageing mainly through oxidative stress mechanisms. Since it is difficult to minimize skin exposure to air pollutants, especially in urban areas, strategies to protect the skin are needed. Plant phenolic compounds have been found to be effective in attenuating cellular oxidative stress and inflammation induced by different air pollutants and a dietary approach based on these compounds could provide an efficient protection measure.

**Objective:**

Here we investigated the efficacy of a commercially available polyphenol-enriched dietary supplement (Zeropollution^®^) in reducing pollution-induced oxidative stress and in improving different skin parameters related to skin ageing of Caucasian and Asian subjects exposed to air pollution. Zeropollution is composed of four standardized herbal extracts: *Olea europaea leaf*, *Lippia citriodora*, *Rosmarinus officinalis*, and *Sophora japonica*.

**Design:**

A double-blind randomized, parallel group study was carried out on 100 outdoor workers living in a polluted urban European area (Milan) to assess the efficacy of the dietary supplement. The total antioxidant capacity on saliva (FRAP), the oxidative damage on skin (lipoperoxides content), skin moisturization (corneometer), transepidermal water loss (tewameter), skin radiance and colour (spectrophotometer), skin elasticity (cutometer), skin sebum content (sebumeter), and the skin roughness (image analysis) were measured.

**Results:**

Both inter-group and intra-group analysis proved that the dietary supplement improved all clinical and biochemical-monitored parameters, in both Caucasian and Asian individuals. Some of the positive effects such as decreased wrinkle depth, increased elasticity and firmness, improved skin moisturization and transepidermal water loss, and reduced dark spots pigmentation were statistically significant as early as 2 weeks of product consumption.

**Conclusions:**

The results of the study indicate reduced oxidative stress-induced skin damage in both Asian and Caucasian women living in a polluted urban area. Therefore, the oral intake of this four-plant based supplement could be considered a complementary nutrition strategy to avoid the negative effects of environmental pollution exposure.

## Popular scientific summary

- Exposure to air pollution results in accelerated skin aging, inflammatory or allergic skin conditions such as atopic dermatitis and acne. Since it is difficult to escape from air pollution it is essential to take measures to protect and repair the skin.
- The polyphenol-enriched dietary ingredient, Zeropollution^®^, has been shown to improve skin ageing signs, to strengthen the skin barrier function, and to counteract the oxidative stress in women living in high pollution urban areas.

Air pollution has become a major problem in recent decades and is viewed as the world’s largest environmental health risk factor, responsible for millions of deaths globally each year. A new WHO air quality model confirms that 92% of the world’s population lives in cities where air quality levels are below healthy limits (1).

The sources of pollution vary from cigarettes, natural sources (such as windblown dust, sea-salt spray or volcanic eruptions) and to a large extent emission from transport and industrial activities. The pollutants of major concern regarding public health are fine particulate matter (PM), defined by a diameter ≤0.1 μm (ultrafine particles [UFP]), ≤2.5 μm (PM 2.5) or ≤10 μm (PM 10); gases, such as nitrogen dioxide (NO_2_), carbon monoxide (CO), volatile organic compounds (VOCs) or ground-level ozone (O_3_) and toxic chemicals, for instance, polycyclic aromatic hydrocarbons (PAH) and dioxins.

Although it is reasonable to assume that the skin is one of the first organs to be affected by air pollution, our knowledge regarding the harmful effects of air pollution on skin physiology remains limited. It has been recently shown that air pollution, mainly PM, can penetrate the skin barrier and alter its redox status (2, 3). Particulate matter induces oxidative stress, increases reactive oxygen species (ROS) production and secretion of pro-inflammatory cytokines, resulting in lipid peroxidation (LPO) and DNA damage. This further increases matrix metalloproteinases, (MMPs)-1, -2, and -9, which degrade collagen (4). Aside from solar radiation (ultraviolet radiation [UVR]), pollution has been recognized as one of the driving factors that accelerates skin ageing, such as pigment spots on face, nasolabial folds, and wrinkles (5). Furthermore, recent studies suggest that the effects of UV radiation and air pollution are not independent of each other, suggesting that facial lentigines are the consequence of an interplay of UVR and traffic air related pollutants (6).

Several mechanisms through which air pollutants cause skin damage and ageing have been proposed. Specifically, the current evidence indicates four potential mechanisms: (a) increased skin oxidative stress, (b) promotion of a proinflammatory environment in the skin and disruption of the skin barrier, (c) activation of the aryl hydrocarbon receptor (AhR), and (d) alteration of the skin microbiome (7).

The damaging effects of air pollution may lead to skin disorders, including sensitive skin, skin dryness, accelerated skin ageing and abnormal pigmentation, among others (8–11). Air pollutants are also involved in the onset of atopic dermatitis, eczema, skin rashes, and skin cancers (12, 13). Therefore, efficient protection of the skin against the air pollution exposure is very important not only for cosmetic reasons.

Topical skincare products are eligible candidates to protect the skin from air pollution. They act upon two major pathways: limiting pollution contact with the skin due to their intrinsic film-forming properties, and by incorporating ingredients with antioxidative properties to minimize the damaging effect of pollutants (14). However, topical solutions do not seem to be sufficient for effective protection against pollution. This is because cosmetics can only protect the most external layers of the skin, while it is known that internal structures of the skin can also be affected. Ultra-fine particles and PAHs may accumulate in the hypodermis, dermis, and bottom of the hair follicle, which are highly vascularized, and can even reach the deep epidermis (15). Moreover, certain pollutants can penetrate the skin via indirect systemic distribution of inhaled or ingested pollution through the blood (15–17). It is for these reasons that the shielding efficacy of skincare products should be complemented with a dietary approach in a more sophisticated skin-protecting strategy. The dietary approach, in fact, could be an adequate candidate to replenish the skin with nutrients that are able to increase the repairing mechanisms from the effect of chronic air pollution exposure. Several clinical trials have shown that supplementation with natural compounds such as vitamins, minerals, plant polyphenols, carotenoids, collagen peptides, etcetera have shown to have anti-ageing and photoprotective effects (18–20). Moreover, there is substantial evidence that including antioxidants in the diet, in the form of fresh foods or through food supplements, may play a role in modulating the acute effects of air pollutants in lung function and cardiovascular system (21, 22).

Extracts and phenolic compounds derived from many plants, such as blueberry, green tea, grape, pomegranate and different marine algae, have been proven to be effective in attenuating the cellular oxidative stress and inflammation induced by different air pollutants (23–28). Published preclinical studies have shown that extracts from *Lippia citriodora leaf, Olea europaea leaf, Sophora japonica, and Rosmarinus officials*, as well as its main bioactive phenolic compounds, are effective antioxidants with the ability to counteract different environmentally induced skin damage, such as UV radiation (29–39). Also, a preliminary and unpublished study of a commercially and patented blend of these four herbal extracts standardized in verbascoside, oleuropein, hydroxytorosol, quercetin and rosemary diterpenes demonstrated significantly antioxidant and anti-inflammatory properties in skin explants exposed to pollutants (40).

Based on all the above findings, in the current study we investigated *in vivo* the efficacy of the test product against the detrimental effects of air pollution on the skin. To this end, a double-blind, placebo-controlled study was performed to evaluate the efficacy of this four-extract blend in improving the skin condition of Caucasian and Asian females living in a homogenous urbanized, highly polluted area and that spend at least 2 h daily outdoors. Milan was chosen as it is one of the most polluted cities in Europe (41).

When assessing active ingredients for their performance against exposure to pollution, safety considerations are of primary importance and exposing humans to pollutants can pose ethical concerns with regard to health hazards. In this sense, testing the product in real-life conditions in volunteers selected, based on their living conditions and lifestyle for example, people who spend a large amount of time in highly polluted areas, are generally recruited to study the antipollution properties of cosmetic ingredients (24, 42, 43).

Here, we demonstrate that the intake of the polyphenolic-enriched blend (*Rosmarinus officinalis, Olea Europaea, Lippia citriodora* and *Sophora japonica*) for 12 weeks significantly improved systemic and skin oxidative status, strengthened the skin barrier, improved skin moisturization, regulated sebum secretion and provided anti-ageing skin benefits, versus the placebo group.

## Materials and methods

### Study design and ethics statement

A monocentric, stratified (50% Asian and 50% Caucasian subjects), randomized, double-blind, placebo-controlled, parallel-group study was conducted in Italy during the winter period.

The study took place at Complife Group facilities in Milan (MI) in accordance with the Declaration of Helsinki (Ethical Principles for Medical Research Involving Human Subjects). Complife Group is an independent testing laboratory for *in vitro* and *in vivo safety* and efficacy assessment of cosmetics, food supplements and medical devices. The study protocol and the informed consent form were approved by the ‘Independent Ethical Committee for Non-Pharmacological Clinical trials’ (ref. 2018/11). Subjects were randomly assigned to the test dietary supplement and placebo group in a 1:1 ratio allocation rate. All subjects provided written, informed consent before initiation of any study-related procedures. No changes to treatment regimen or methods were necessary after the study initiation.

### Subjects

Subjects were first screened in the volunteer’s database (keywords: Sex ‘female’, Age ‘35–65’, Ethnicity ‘Asian and Caucasian’ Skin type ‘normal and sensitive’, Testing preferences: ‘food supplements’). Eligible participants were then screened by a board-certified dermatologist. During the screening visit, a physical examination was carried out in order to assess the skin conditions. Subjects meeting the inclusion criteria were then enrolled and randomised. Inclusion criteria included adults, Asian and Caucasian (1:1 ratio) female subjects, aged between 35 and 65 years old, with normal or sensitive skin (1:1 ratio), dark spots on the face, that spend at least 2 h outdoors every day in an urbanized area. Subjects were of general good health without any alimentary and/or eating disorders (i.e. bulimia, psychogenic eating disorders, etc.). Exclusion criteria included pregnancy or intention to become pregnant, lactation, food intolerances/allergy, pharmacological treatments and/or food supplement intake known to interfere with the test product or influencing metabolism, participation in another dietary supplement study, unwillingness or inability to comply with the requirements of the study protocol. The study further excluded subjects using products known to interfere with product efficacy and/or with the study outcomes and subjects not willing to refrain from sun exposure if not properly protected. Throughout the study period, subjects were asked to only use the supplied face cream and soap.

### Intervention

The test item was a patented (WO/2019/211501) commercially available food supplement ingredient (Zeropollution^®^, supplied by Monteloeder S.L., Miguel Servet 16, Elche, Alicante, Spain) containing four polyphenolic extracts: *Rosmarinus officinalis* leaf extract standardized in diterpenes, *Olea Europaea* leaf extract standardized in oleuropein and hydroxytyrosol, *Lippia citriodora* leaf extract standardized in verbascoside and *Sophora japonica* extract standardized in quercetin. In total, w/w this blend comprises a minimum content of the following phenols: diterpenes (sum of carnosic acid and carnosol) 4.5%; oleuropein 4.5%; hydroxytyrosol 1.5%; verbascoside 6.5%; and flavones as quercetin minimum 3.5%. These main compounds are identified and quantified by HPLC-DAD analysis, comparing retention time and UV spectra of the peaks in samples with those of authentic standards (more detailed can be seen in the supplementary section). Each capsule contained 250 mg active ingredient and 120 mg microcrystalline cellulose. The placebo product contained 250 mg maltodextrin and 120 mg microcrystalline cellulose. To enhance study blindness, both the placebo and active capsules colour was adjusted with FD&C Blue 2 and titanium dioxide. After the initial visit, subjects started taking one capsule of the dietary supplement or the placebo product every day for 12 weeks. The product intake was 30 min before breakfast. To standardize the daily cosmetic routine, subjects were supplied with a neutral day/night cream and a cleansing lotion.

### Primary and secondary outcomes

The primary endpoints concerning the efficacy in protecting the skin against environmental pollutants were the measurement of the total antioxidant capacity in the saliva and the oxidative damage of the skin. The total antioxidant capacity in the saliva were measured at baseline and after 4 and 12 weeks of the intervention period and the oxidative damage of the skin were measured at the beginning and after 4, 8, and 12 weeks of treatment.

Skin moisturization, sebum content, elasticity, colour, radiance and roughness analysis were measured as secondary endpoints and were taken at baseline, and after 2, 4, 8 and 12 weeks of the intervention period. A self-assessment efficacy evaluation questionnaire was also performed after 1 month and at the end of the study to evaluate the perceived efficacy by the subjects.

### Measurement of skin lipoperoxides

Basal skin lipoperodixes (LPO) were measured in the 10^th^ skin layer obtained using the skin stripping technique. Skin stripping was performed in the back using Corneofix^®^ foils (Courage+Khazaka Electronic, Köln, Germany) under standard pressure conditions (225 g/cm^2^). The first stripping was discarded while strip number 10 was collected and stored at −80°C until further analysis. Malondialdehyde (MDA) was measured according to the assay described by Erdelmeier et al. in 1998 (18, 44) with minor modification (18), as follows: (1) skin strippings were layered in 12 multiwell plates containing 500 μL of a 0.5 mM CuSO_4_ aqueous solution, (2) multiwell plates were incubated at 37°C, using a microplate incubator/shaker under continuous agitation for 1 h, (3) after incubation 1.3 mL R1 solution (2.13 mg N-methyl-2-phenylindole/mL acetonitrile) and 0.3 mL 37% HCl was added and samples were further incubated at 45°C for 60 min under continuous agitation, (4) the reaction was stopped in ice for 10 min followed by 10 min at room temperature, (5) 1 mL of solution was centrifuged at 13.000 rpm per 10 min, and (6) absorbance was read at 586 nm using a multiwell plates reader (programmable MPT reader model DV 990BV6; Gio DeVita & C, Rome, Italy).

### Measurement of total antioxidant capacity of saliva

The total antioxidant capacity of saliva (TAS) was measured using the ferric reducing antioxidant power technique (FRAP) described by Benzie et al. in 1996 (45). Ferric reducing antioxidant power technique is based on the reduction of a ferric-tripyridyltriazine (FeIII-TPTZ) complex to the ferrous (FeII) form at a low pH value. The ferric to ferrous reduction causes a blue-coloured ferrous-tripyridyltriazine complex to form. Briefly, 30 μL of distilled water, 160 μL of working FRAP reagent (25 mL acetate buffer, 2.5 mL TPTZ solution and 2.5 mL FeCl_3_ 6H_2_O solution) and 10 μL saliva sample were added in a 96 multiwell plate. The samples were then incubated at 37°C using a microplate incubator/shaker with 30 min of continuous agitation. Absorbance was read at 595 nm.

### Measurement of skin moisturization

Skin moisturization was measured using a Corneometer^®^ CM 825 (Courage+Khazaka Electronic, Köln, Germany). Corneometer^®^ probe measures the capacitance of the stratum corneum using an electric scatter field penetrating the first layers of the stratum corneum (10–20 μm). The capacitance variation of the probe capacitor due to skin surface hydration was measured and skin moisturization was reported in arbitrary units (corneometric units). The measurement was taken in five different points of the right cheek. The selected measurement points delineate the vertices and the centre of a quadrangle virtually drawn across the cheek. An increase in the Corneometer value is indicative of a skin-moisturizing effect.

### Measurement of wrinkle depth and skin roughness

Wrinkle depth and skin roughness (Ra parameter) were measured using a three-dimensional (3-D) microtopography imaging system (PRIMOS 3D lite, GFMesstechnik GmbH, Teltow, Germany). The imaging system projects structured light on a specific surface of the skin with a digital micro-mirror device (DMD, Texas Instruments, Irving, TX, USA) and records the image with a CCD camera. Skin surface microtopography is then reconstructed using temporal phase shift algorithms to generate 3-D images. The imaging system has an overlap feature which enables precise matching of photos taken at different visits. In order to improve image overlap, the subjects’ position was regulated using a stereotactic device (Canfield Scientific, Inc., Fairfield, NJ, USA). Wrinkle depth and skin roughness were measured in the periocular area (‘crow’s feet wrinkles’) using the appropriate software routine. Ra parameter is related to skin smoothness and a decrease of Ra can be expressed in absolute values as an increase in skin smoothness.

### Measurement of transepidermal water loss

Transepidermal water loss (TEWL, *perspiratio insensibilis*) was measured using a Tewameter^®^ TM 300 (Courage+Khazaka Electronic, Köln, Germany). Tewameter^®^ probe measures indirectly the density gradient of water evaporation over the skin surface using two pairs of sensors (temperature and relative humidity) in an ‘open chamber’ configuration mode. The Fick diffusion law is the basis for the measurement allowing us to calculate the evaporation rate in g·h^-1^·m^-2^. The measurement was taken in the centre of the right cheek. Precautions were taken in order to avoid any turbulence in the measurement area. According to the technical Guide, the scale is as follows: 0–10 (very healthy conditions), 10–15 (healthy conditions), 15–25 (normal condition), 25–30 (affected skin) and >30 (critical condition).

### Measurement of skin sebum content

Skin sebum content, in the centre of the forehead, was measured using a Sebumeter^®^ SM 815 (Courage+Khazaka Electronic, Köln, Germany). Sebumeter^®^ measurement is based on ‘greasy spot photometry’. When the mat tape of the Sebumeter^®^ cartridge is brought into contact with skin (over a 64 mm^2^ surface) it becomes transparent according to the skin sebum content. The transparency (light transmission) of the mat tape is measured by a photocell allowing us to calculate the skin sebum content in μg/cm^2^.

### Measurement of skin elasticity and firmness

Skin elasticity was measured using a Cutometer^®^ MPA 580 (Courage+Khazaka Electronic, Köln, Germany) skin viscoelasticity analyser. The skin surface of the face (right cheek) was drawn into the aperture (3 mm) of the probe by a negative pressure (450 mbar) for 3 sec and thereafter released for 3 sec. The penetration depth of the skin inside the probe, during the suction and the release phase was measured by a non-contact optical measuring system. R0 and R2 elasticity parameters were measured. R0 is known as the first maximum amplitude of the first suction curve (Uf, skin distensibility), representing the passive behaviour of the skin to force. R0 is linked with the stretching of both collagen and elastic fibres and is inversely proportional to their thickness and rigidity. R2 is known as the skin ‘gross’ or ‘overall’ elasticity and is represented by the ratio between the ‘residual deformation’ (Ua) and its maximum ‘distensibility’ (Uf). R2 is a relative elasticity parameter widely used to assess skin elasticity and ageing.

### Measurement of skin colour (melanin content) and skin radiance

Skin melanin content was indirectly evaluated by measuring skin colour components related to melanin content by colorimetry. The individual typology angle (ITA°) by means of L* and b* skin colour measurement using a Colourimeter CM-700D (Konica Minolta, Milan, Italy) was calculated. Individual typology angle measurement is based on the measurement of L* and b* colour components measurement by a skin colourimeter. Individual typology angle was then calculated as follows: ITA = [ArcTan((L*−50)/b*)]x180/π. Based on ITA° values, skin colour can be classified as follows: ITA > 55° very light skin colour, 55 ≥ ITA > 41 light skin colour, 41 ≥ ITA > 28 intermediate skin colour, 28 ≥ ITA > 10 tan/matt skin colour. Individual The more ITA° increases, the lighter the skin.

Skin radiance was evaluated by measuring the gloss values with a Colourimeter CM-700D (Konica Minolta, Milan, Italy). The d:8° geometry of the colourimeter features an optical device providing diffuse illumination (Ulbricht sphere). The light (Xenon lamp) is projected into a sphere coated with a white highly reflecting substance (barium sulphate, ceramic, special plastic) which reflects the light manifold. A shutter inside the sphere prevents the directional rays from reaching the measuring sample directly. The skin area to be measured is positioned at an opening of the sphere and is illuminated from all directions with a close to perfect diffuse light. The sensor views the surface being measured through an opening at the top of the sphere with an angle of 8° to the vertical axis. In order to prevent the reflection of specular light from the sample surface, the instrument features a gloss trap. When the trap, which is arranged with an angle of −8° to the viewing opening, is opened, the light which would otherwise be reflected from the interior wall of the sphere is eliminated and can therefore not illuminate the sample. The relation between directional and diffuse reflection allows calculating the gloss component. The measurement was taken in the cheek.

### Subjective self-assessment of the efficacy of the product

The subject-based evaluation of the efficacy of the tested product was performed using a 12-item questionnaire adapted to the investigated product and completed by all the study participants at week 4 and at the end of the study (week 12). For each item, answers were recorded on a 4-point grading scale (completely agree, agree, disagree and completely disagree) and results were expressed as the percentage of subjects in agreement.

### Sample size

The sample size was calculated, for the long-term study, with a two-sided 5% significance level and a power of 80% taking into account a 20% variation of the primary endpoints due to both inter-individual human variability and error in the measurement techniques. Sample size was calculated using PASS 11 statistical software (version 11.0.8 for Windows) running on Windows Server 2008 R2 Standard SP1 64-bit edition (Microsoft, USA). A sample size of 20 subjects per group was necessary given an anticipated dropout rate of 20%.

### Randomisation

A restricted randomisation list was created using PASS 11 (version 11.0.8; PASS, LLC. Kaysville, UT, USA) statistical software running on Windows Server 2008 R2 Standard SP1 64-bit edition (Microsoft, USA) by a biostatistician and stored in a safe place. The randomisation sequence was stratified using ‘Efron’s biased coin’ algorithm with a 1:1 allocation ratio. The allocation sequence was concealed from the study director in sequentially numbered, opaque and sealed envelopes, reporting the unblinded treatment allocation (based on subject entry number in the study). The A4 sheet reporting the unblinded treatment was folded to render the envelope impermeable to intense light. After acceptance of the subject in the study, the appropriate numbered envelope was opened. An independent technician dispensed either dietary supplement or placebo products according to the card inside the envelope. The study adhered to established procedures to maintain separation between the investigator and its collaborators and the staff that delivered the intervention. Staff who delivered the intervention did not take outcome measurements. Subjects, investigators and collaborators were kept masked to product assignment. The dietary supplement and placebo products were in capsule form and identical in appearance. They were pre-packed in blisters and consecutively numbered for each subject according to the randomisation schedule. Each subject was assigned an order number and received the capsules in the corresponding pre-packed blister.

### Safety

The occurrence of adverse events (AEs) was monitored throughout the study by the investigators and based on subjects’ diary entries. Investigators rated the observed and reported AEs as being either severe or non-severe based upon their potential relationship to study treatment.

### Statistical methods

Statistical analysis was performed using NCSS 8 (version 8.0.4 for Windows; NCSS, Kaysville, UT, USA) running on Windows Server 2008 R2 Standard SP1 64-bit edition (Microsoft, USA). Data normality was checked using Shapiro–Wilk W normality test and data shape. Both intragroup (vs. baseline) and intergroup (between treatments) statistical analysis was carried out two-way t test of student. A *P* < 0.05 was considered statistically significant. Statistical analysis output was reported as follows: *P < 0.05, **P < 0.01, and ***P < 0.001.

## Results

**Fig. 1:**
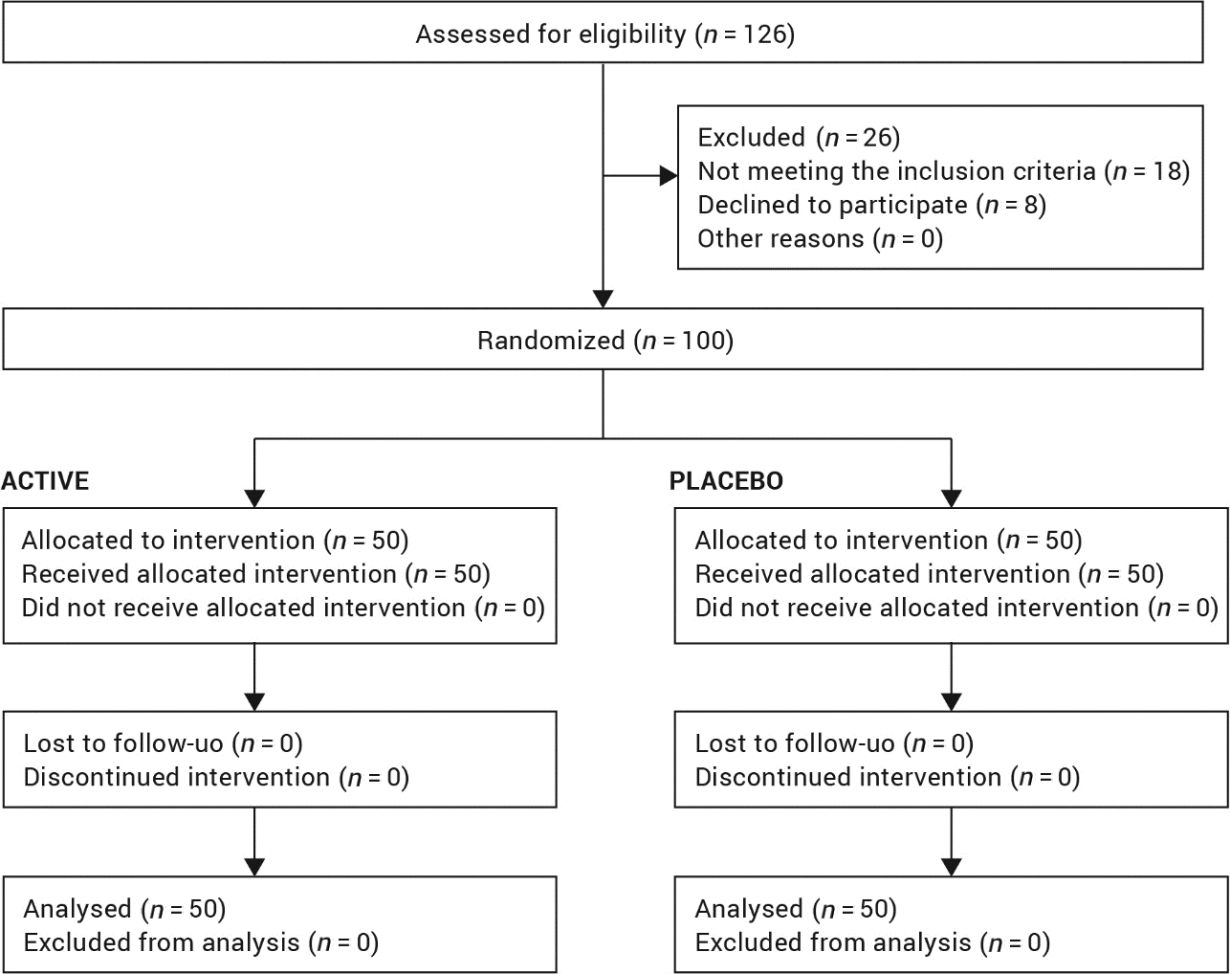
Flow chart of inclusion of subjects.

A total of 100 female subjects were successfully randomised and all of them completed the full course of treatment and follow-up (Fig. 1). Data analysis was intention-to-treat and involved all subjects who were randomly assigned. Subjects’ compliance to treatment was assessed through product accountability, as follows: at each visit, the expected amount of consumed capsule was compared with the amount dispensed minus the amount the subject returned. No major deviation was observed in the treatment regimen. All subjects were included in the safety analysis data set. Subjects attended clinic visits at the time of randomisation (baseline) and after 2, 4, 8, 12 weeks of product use. All the tested products were well tolerated. No adverse reactions occurred during the study period.

Demographic and baseline characteristics (Table 1) were similar across treatment arms, indicating unbiased randomisation and the absence of covariates. The mean age of volunteers in the study was 50.6 ± 1.0 years, 35 years old being the minimum age and the maximum age 65 years old. Regarding the gender, 100% of participants were women. The population was 50% Caucasian and 50% Asian and the 50% of them had sensitive skin type based on lactic acid stinging test.

**Table 1:** Demographic and baseline characteristics of study participants

	Treatment group			Placebo group		
	Asian	Caucasian	Overall	Asian	Caucasian	Overall
Sex						
Male (*N*)	0	0	0	0	0	0
Female (*N*)	25	25	50	25	25	50
Age (years)	48.3 ± 1.1	52.7 ± 1.3	50.5 ± 0.9	49.7 ± 1.2	51.9 ± 1.6	50.8 ± 1.0
Sensitive skin (%)	48	52	50	52	48	50
Smokers (%)	4	20	12	0	16	8
Skin moisturization (a.u)	46.2 ± 2.1	34.4 ± 1.4	40.3 ± 1.5	40.7 ± 1.8	37.7 ± 1.6	39.2 ± 1.2
TEWL (g·h^-1^·m^-2^)	17.6 ± 1.0	11.3 ± 0.5	14.5 ± 0.7	17.7 ± 0.9	11.9 ± 0.5	14.8 ± 0.6
Wrinkle depth (μm)	269.7 ± 19.6	299.7 ± 20.9	284.7 ± 14.3	252.4 ± 13.8	270.7 ± 23.1	261.6 ± 13.4
Ra (μm)	27.9 ± 1.4	30.7 ± 1.0	29.3 ± 0.9	27.2 ± 1.6	30.3 ± 0.9	28.8 ± 0.9
R2 (Ua/Uf)	0.5598 ± 0.0136	0.6686 ± 0.0135	0.6142 ± 0.0123	0.5419 ± 0.0151	0.6208 ± 0.0190	0.5814 ± 0.0133
R0 (Uf) (mm)	0.3279 ± 0.0190	0.3500 ± 0.056	0.3389 ± 0.0099	0.3491 ± 0.0130	0.3578 ± 0.0092	0.3535 ± 0.0079
ITA° (°)	42.0 ± 1.7	31.9 ± 1.4	37.0 ± 1.3	43.0 ± 1.5	32.2 ± 1.3	37.6 ± 1.2
Skin radiance (a.u)	5.7 ± 0.3	10.8 ± 0.3	8.2 ± 0.4	6.3 ± 0.2	11.6 ± 0.4	8.9 ± 0.5
Sebum content (μg/cm^2^)	100.9 ± 4.5	153.8 ± 13.4	127.4 ± 8.0	110.6 ± 4.1	174.3 ± 14.0	142.5 ± 8.5
MDA (μM)	2.7 ± 0.1	2.5 ± 0.1	2.6 ± 0.1	2.7 ± 0.1	2.5 ± 0.1	2.6 ± 0.1
TAS (μM Fe^2+^)	446.5 ± 12.4	430.5 ± 16.6	438.5 ± 10.3	440.3 ± 11.2	419.2 ± 18.7	429.7 ± 10.9

Values are means ± standard error (SE).

Throughout the study period, environmental conditions (temperature, UV radiation, humidity) and air quality (PM 10, PM 2.5, S0_2_, N0_2_ O_3_ and benzene) were recorded. During the days when the clinical trial was conducted, elevated PM10 and PM2.5 concentrations in the respective urban areas were registered (Agenzia Regionale per la Protezione dell’ambiente della Lombardia). Study population were exposed to PM10 air pollutant concentrations above WHO air quality guidelines (>20 μg/m^3^) during 90% of the study time and above 50 μg/m^3^ during 36% of the time. The exposure to fine PM (2.5) was even worse, surpassing the WHO healthy limits (10 μg/m^3^) 96% of the time and above the 25 μg/m^3^ limit 60% of the time. The PM 10 and PM 2.5 average during the period was 45 ± 21 μg/m^3^ and 35 ± 21 μg/m^3^, respectively. The daily mean value of the major air quality parameters, throughout the study period, can be seen in the supplementary section (Supplementary Fig. 2).

**Fig. 2:**
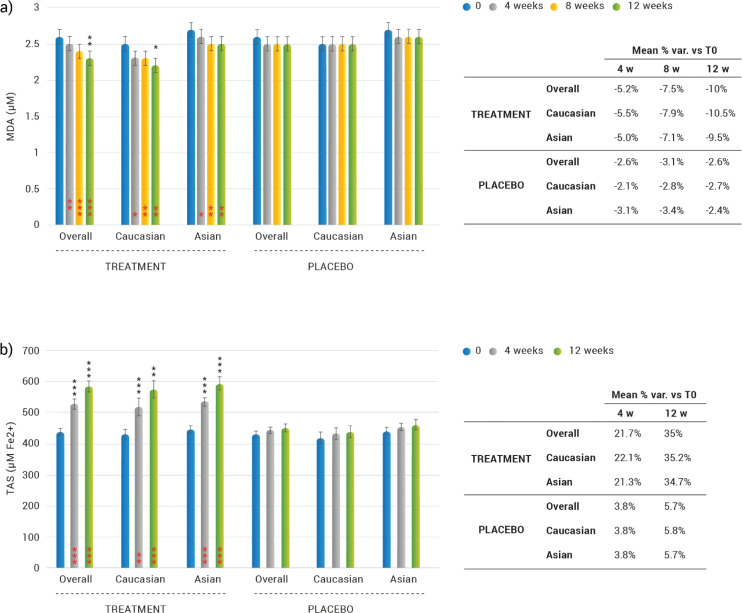
Skin and systemic oxidative status after 2, 4, 8, and 12 weeks of treatment. (a) Lipid-peroxidation (LPO) levels measured by MDA content on skin cells. Data are means (μM) ±SEM. (b) Total antioxidant capacity of saliva (TAS) measured by FRAP technique. Data are means (μM Fe2+) ±SEM. Intragroup (vs. 0) statistical analysis is reported inside the bars of the histograms (in red). Intergroup (vs. placebo) statistical analysis is reported upon the bars of the histograms. Statistical analysis is reported as follows: *P < 0.05, **P < 0.01, and ***P < 0.001. In the tables are reported the percentage variation versus baseline.

### Effects on skin and systemic oxidative status

#### Skin LPO through MDA measurement

A statistically significant decrease of the basal LPO content was observed both in Caucasian and Asian subjects (Fig. 2a). The overall MDA content in the skin horny layer was decreased by 5.2 (*P* = 0.0025), 7.5 (*P <* 0.0001) and 10.0% (*P <* 0.0001), after 4, 8, and 12 weeks of treatment with the dietary supplement; respectively. In the subpopulations, the MDA content decreased in Caucasian subjects by 5.5 (*P* = 0.0350), 7.9 (*P* = 0.0042) and 10.5% (*P* = 0.0026), after 4, 8, and 12 weeks treatment; respectively. As for the Asian volunteers, MDA content decreased by 5.0 (*P* = 0.0350), 7.1 (*P* = 0.0042) and 9.5% (*P* = 0.0026), after 4, 8, and 12 weeks treatment; respectively. No statistically significant differences were observed when comparing Asian and Caucasian subjects. Statistical significance in the inter-group analysis of MDA content was only detected at the end of the study in the whole study population (Asian and Caucasian subjects) (*P* = 0.0047) and in the Caucasian subjects (*P* = 0.0463). On the other hand, MDA content was unchanged in the placebo-treated subjects throughout the study.

#### Total antioxidant capacity of saliva

A statistically significant increase of TAS was observed both in Caucasian and Asian subject’s treatment group (Fig. 2b). The overall (Caucasian and Asian subjects) increase in TAS was by 21.7 (*P* = 0.0025) and 35.0% (*P* < 0.0001) after 4 and 12-weeks treatment, respectively. Total antioxidant capacity of saliva increase in Caucasian subjects was by 22.1 (*P* = 0.0020) and 35.2% (*P* < 0.0001) after 4 and 12 weeks, respectively. Total antioxidant capacity of saliva increase in Asian subjects was by 21.3 (*P* < 0.0001) and 34.7% (*P* < 0.0001) after 4 and 12 weeks, respectively. No statistically significant differences were observed between Asian and Caucasian subjects in the % variation. The TAS variation observed in the dietary supplement group was statistically significant when compared to the placebo treatment regimen, both overall (Caucasian and Asian subjects) and when considering only Caucasian or Asian subjects, at all the investigated checkpoints. However, TAS was unchanged in the placebo-treated subjects throughout the study.

### Effects on facial skin condition

#### Transepidermal water loss

Changes in the epidermal barrier function were evaluated by measuring the TEWL. A statistically significant decrease of TEWL was observed in all the treatment groups (Table 2). The overall (Caucasian and Asian subjects) TEWL decrease was by 3.8 (*P* = 0.0001), 6.2 (*P* < 0.0001), 9.2 (*P* < 0.0001) and 10.1% (*P* < 0.0001) after 2, 4, 8, and 12 weeks of treatment, respectively. Transepidermal water loss decrease in Caucasian subjects was by 3.5 (*P* = 0.0011), 5.2 (*P* = 0.0001), 8.7 (*P* < 0.0001) and 8.6% (*P* < 0.0001) after 2, 4, 8, and 12 weeks of treatment, respectively. Transepidermal water loss decrease in Asian subjects was by 4.1 (*P* = 0.0099), 7.3 (*P* < 0.0001), 9.7 (*P* = 0.0001) and 11.6% (*P* < 0.0001) after 2, 4, 8, and 12 weeks of treatment, respectively. Although the Asian panel appears to have a greater decrease in TEWL at the end of the study, the differences are not statistically significant compared to the Caucasian group. The TEWL variation observed in the treatment group was statistically significant, when compared to the placebo, both overall (Caucasian and Asian subjects) at all the investigated checkpoints and when only considering Caucasian or Asian subjects, starting from 4 weeks product use. In the placebo group a slight decrease of the TEWL was observed (−2.3% at the end of the study).

**Table 2:** Effects on Transepidermal water loss (TEWL) and on skin moisture (water content) in facial skin

Measurement	Groups		Intervention period				
			0 weeks	2 weeks	4 weeks	8 weeks	12 weeks
TEWL (g/m^2^/h)	Treatment	Overall	14.5 ± 0.7	13.9 ± 0.7*** (−3.8%)ᶴ	13.5 ± 0.6*** (−6.2%)ᶴ	13.0 ± 0.6*** (−9.2%)ᶴ	12.8 ± 0.6*** (−10.1%)ᶴ
		Caucasian	11.3 ± 0.5	10.9 ± 0.4** (−3.5%)	10.7 ± 0.4*** (−5.2%)ᶴ	10.2 ± 0.4*** (−8.7%)ᶴ	10.2 ± 0.3*** (−8.6%)ᶴ
		Asian	17.6 ± 1.0	17.0 ± 1.0*** (−4.1%)	16.3 ± 0.9*** (−7.3%)ᶴ	15.7 ± 0.8*** (−9.7%)ᶴ	15.4 ± 0.8*** (−11.6%)ᶴ
	Placebo	Overall	14.8 ± 0.6	14.5 ± 0.6* (−1.5%)	14.3 ± 0.6*** (−2.8%)	14.3 ± 0.6** (−2.7%)	14.4 ± 0.6** (−2.3%)
		Caucasian	11.9 ± 0.5	11.7 ± 0.4** (−1.8%)	11.6 ± 0.4*** (−2.6%)	11.6 ± 0.4*** (−2.6%)	11.5 ± 0.4*** (−3.3%)
		Asian	17.7 ± 0.9	17.4 ± 0.8 (−1.3%)	17.0 ± 0.8** (−3.1%)	17.0 ± 0.7* (−2.8%)	17.7 ± 0.7 (−1.4%)
Water Content (a.u.)	Treatment	Overall	40.3 ± 1.5	42.5 ± 1.6*** (+5.7%)ᶴ	43.7 ± 1.6*** (+9.0%)ᶴ	44.7 ± 1.5*** (+12.3%)ᶴ	45.6 ± 1.4*** (+14.8%)ᶴ
		Caucasian	34.4 ± 1.4	36.0 ± 1.3** (+5.0%)ᶴ	36.8 ± 1.3*** (+7.4%)ᶴ	38.0 ± 1.2*** (+11.4%)ᶴ	39.1 ± 1.1*** (+15.1%)ᶴ
		Asian	46.2 ± 2.1	49.0 ± *2.3*** (+6.5%)ᶴ	50.7 ± 2.1*** (+10.6%)ᶴ	51.4 ± 1.9*** (+13.2%)ᶴ	52.1 ± 1.9*** (+14.5%)ᶴ
	Placebo	Overall	39.2 ± 1.2	39.4 ± 1.1 (+1.2%)	40.4 ± 1.2*** (+3.5%)	41.2 ± 1.2*** (+5.8%)	41.0 ± 1.1*** (+5.3%)
		Caucasian	37.7 ± 1.6	38.5 ± 1.6 *** (+2.3%)	39.0 ± 1.6*** (+3.8%)	39.3 ± 1.6*** (+4.5%)	39.3 ± 1.5*** (+4.8%)
		Asian	40.7 ± 1.8	40.3 ± 1.6 (+0.0%)	41.7 ± 1.7 (+3.3%)	43.2 ± 1.7*** (+7%)	42.7 ± 1.6*** (+5.8%)

Data are mean ± SEM. In brackets is reported the percentage variation versus baseline. Intragroup (vs. baseline) statistical analysis is reported as follows:

* *P* < 0.05,

** *P* < 0.01, and

*** *P* < 0.001.

ᶴ Statistically different versus placebo.

#### Skin moisturization (water content)

A statistically significant increase of the skin moisture content was observed both in Caucasian and Asian subjects (Table 2). The overall (Caucasian and Asian subjects) skin moisturization increase was by 5.7 (*P* = 0.0001), 9.0 (*P* < 0.0001), 12.3 (*P* < 0.0001) and 14.8% (*P* < 0.0001) after 2, 4, 8, and 12 weeks treatment, respectively. Skin moisturization increase in Caucasian subjects was by 5.0 (*P* < 0.0001), 7.4 (*P* < 0.0001), 11.4 (*P* < 0.0001) and 15.1% (*P* < 0.0001) after 2, 4, 8, and 12 weeks of treatment, respectively. Skin moisturization increase in Asian subjects was by 6.5 (*P* = 0.0067), 10.6 (*P* < 0.0001), 13.2 (*P* < 0.0001) and 14.5% (*P* < 0.0001) after 2, 4, 8 and 12 weeks of treatment, respectively. No statistically significant differences were observed between Asian and Caucasian subjects. A slight increase in water content was observed in the placebo group at the end of the study (5.3%), however the skin moisturization variation observed in the dietary supplement group was significantly higher when compared to the placebo treatment regimen, both overall (Caucasian and Asian subjects) and for Caucasian and Asian subjects, at all the investigated checkpoints.

### Effect on skin texture parameters

#### Wrinkle depth

**Fig. 3:**
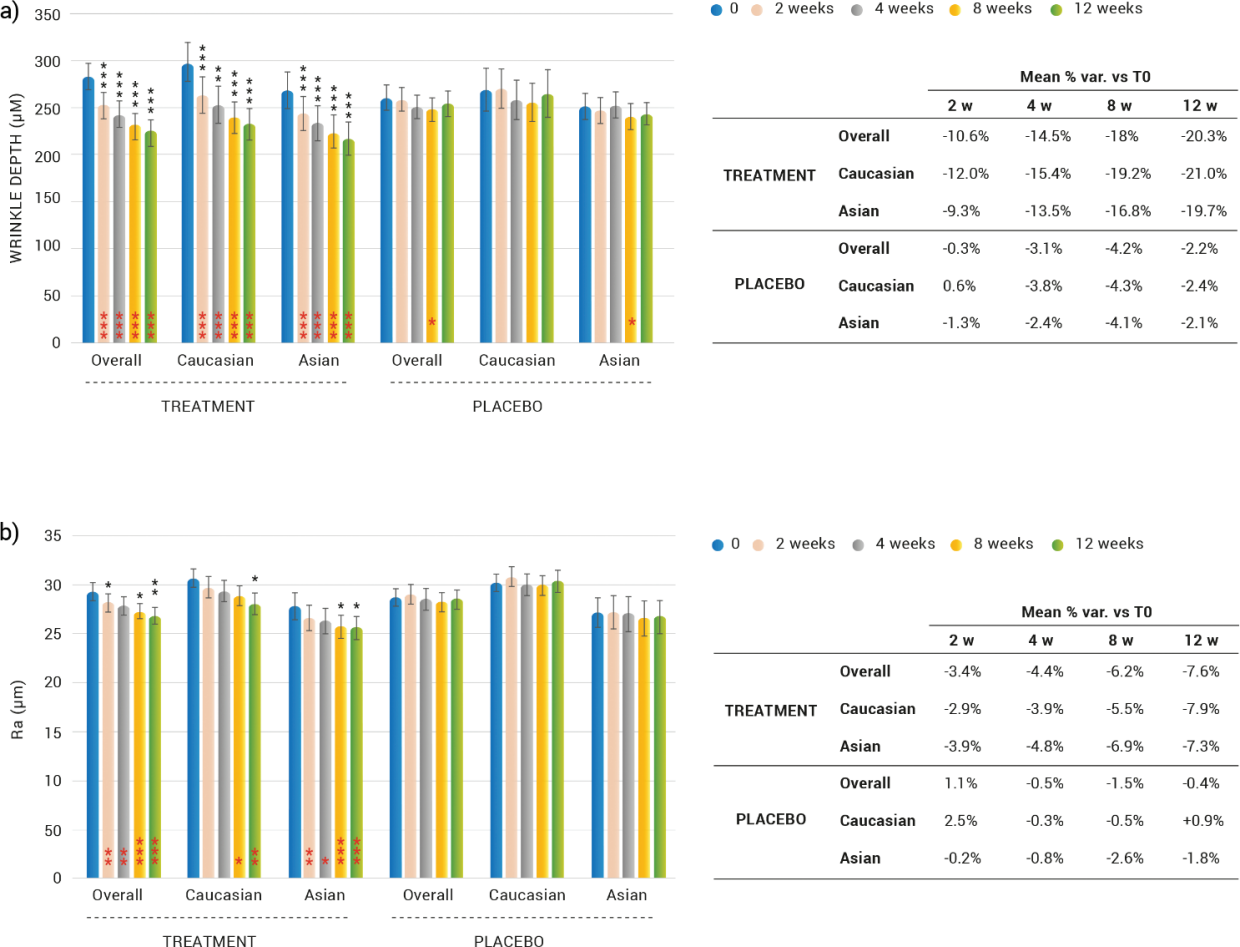
Change on facial skin texture after 2, 4, 8, and 12 weeks of treatment. (a) Wrinkles depth variation. (b) Skin roughness (Ra parameter). Intragroup (vs. 0) statistical analysis is reported inside the bars of the histograms. Intergroup (vs. placebo) statistical analysis is reported upon the bars of the histograms. Statistical analysis is reported as follows: *P < 0.05, **P < 0.01, and ***P < 0.001. Data are means (μm) ±SEM. In the tables are reported the percentage variation versus baseline.

A statistically significant decrease of wrinkle depth in the ‘crow’s feet’ was observed both in Caucasian and Asian subjects taking the dietary supplement (Fig. 3a) from week 2 onwards. The overall (Caucasian and Asian subjects) wrinkle depth decrease was by 10.6 (*P* < 0.0001), 14.5 (*P* < 0.0001), 18.0 (*P* < 0.0001) and 20.3% (*P* < 0.0001) after 2, 4, 8 and 12 weeks of treatment, respectively. Wrinkle depth decrease in Caucasian subjects was by 12.0 (*P* = 0.0005), 15.4 (*P* < 0.0001), 19.2 (*P* < 0.0001) and 21.0% (*P* < 0.0001) after 2, 4, 8 and 12 weeks of treatment, respectively. Wrinkle depth decrease in Asian subjects was by 9.3 (*P* < 0.0001), 13.5 (*P* < 0.0001), 16.8 (*P* = 0.0001) and 19.7% (*P* < 0.0001) after 2, 4, 8 and 12 weeks of treatment, respectively. No statistically significant differences were observed between Asian and Caucasian subjects’ improvement. The wrinkle depth variation observed in the dietary supplement group was statistically significant when compared to the placebo treatment regimen, both overall (Caucasian and Asian subjects) and for Caucasian and Asian subjects, at all the investigated checkpoints. Wrinkle depth was unchanged (*P* > 0.05) in the placebo-treated subjects throughout the study.

#### Skin roughness

A statistically significant decrease of skin roughness (Ra parameter) was observed both in Caucasian and Asian subjects (Fig. 3b). The overall (Caucasian and Asian subjects) skin roughness decrease was by 3.4 (*P* = 0.0067), 4.4 (*P* = 0.0022), 6.2 (*P* < 0.0001) and 7.6% (*P* < 0.0001) after 2, 4, 8 and 12 weeks of treatment, respectively. Skin roughness decrease in Caucasian subjects was by 2.9 (*P* = 0.1680), 3.9 (*P* = 0.0547), 5.5 (*P* = 0.0131) and 7.9% (*P* = 0.0041) after 2, 4, 8 and 12 weeks of treatment, respectively. Skin roughness decrease in Asian subjects was by 3.9 (*P* = 0.0043), 4.8 (*P* = 0.0180), 6.9 (*P* = 0.0002) and 7.3% (*P* = 0.0003) after 2, 4, 8 and 12 weeks of treatment, respectively. No statistically significant differences were observed between Asian and Caucasian subjects. The skin roughness variation observed in the active treatment group was statistically significant when compared to the placebo regimen, as follows: overall (Caucasian and Asian subjects) group after 2, 8 and 12 weeks; for Asian subjects the variation was statistically significant starting from the eighth week of product use; while for Caucasian subjects the variation was statistically significant after 12 weeks of product use. No variations were monitored (*P* > 0.05) in the placebo-treated subjects throughout the study.

### Effects on skin elasticity

Results showed that the overall skin firmness and elasticity improved in the course of the study in the treatment group (Table 3).

**Table 3:** Effects on skin elasticity

Measurement	Groups		Intervention period				
			0 weeks	2 weeks	4 weeks	8 weeks	12 weeks
R0 (mm)	Treatment	Overall	0.3389 ± 0.0099	0.3259 ± 0.0095*** (−3.8%)ᶴ	0.3224 ± 0.0091*** (−4.7%)ᶴ	0.3157 ± 0.0090 *** (−6.7%)ᶴ	0.3143 ± 0.0092*** (−7.1%)ᶴ
		Caucasian	0.3500 ± 0.0056	0.3394 ± 0.0053*** (−3.0%)ᶴ	0.3356 ± 0.0054*** (−4.1%)ᶴ	0.3284 ± 0.0051*** (−6.1%)ᶴ	0.3560 ± 0.0050*** (−6.8%)ᶴ
		Asian	0.3279 ± 0.0190	0.3124 ± 0.0179*** (−4.5%)	0.3092 ± 0.0172*** (−5.3%)ᶴ	0.3031 ± 0.0170*** (−7.2%)ᶴ	0.3027 ± 0.0175*** (−7.3%)ᶴ
	Placebo	Overall	0.3535 ± 0.0079	0.3495 ± 0.0078 (−0.9%)	0.3524 ± 0.0078 (−0.1%)	0.3541 ± 0.0081 (+0.3%)	0.3497 ± 0.0080 (−1.0%)
		Caucasian	0.3578 ± 0.0130	0.3535 ± 0.0092*** (−1.2%)	0.3506 ± 0.0089*** (−2.0%)	0.3494 ± 0.0088*** (−2.3%)	0.3488 ± 0.0088*** (−2.5%)
		Asian	0.3491 ± 0.0130	0.3454 ± 0.0127 (−0.6%)	0.3543 ± 0.0129 (+1.8%)	0.3588 ± 0.0137 (+2.9%)	0.3507 ± 0.0136 (+0.5%)
R2 (Ua/Uf)	Treatment	Overall	0.6142 ± 0.0123	0.6303 ± 0.0121*** (+2.9%)ᶴ	0.6485 ± 0.0108*** (+6.1%)ᶴ	0.6550 ± 0.0110*** (+7.1%)ᶴ	0.6601 ± 0.0112*** (+8.0%)ᶴ
		Caucasian	0.6686 ± 0.0135	0.6888 ± 0.0128*** (+3.1%)	0.7025 ± 0.0100*** (+5.5%)	0.7101 ± 0.0104*** (+6.6%)	0.7171 ± 0.0106*** (+7.6%)
		Asian	0.5598 ± 0.0136	0.5718 ± 0.0124 (+2.6%)	0.5944 ± 0.0114*** (+6.8%)ᶴ	0.5999 ± 0.0116*** (+7.7%)ᶴ	0.6030 ± 0.0114*** (+8.3%)ᶴ
	Placebo	Overall	0.5814 ± 0.0133	0.5872 ± 0.0138* (+0.9%)	0.5847 ± 0.0150 (+0.3%)	0.5831 ± 0.0147 (+0.1%)	0.5872 ± 0.0142 (+0.9%)
		Caucasian	0.6208 ± 0.0190	0.6309 ± 0.0188 *** (+1.7%)	0.6359 ± 0.0186*** (+2.6%)	0.6394 ± 0.0179*** (+3.2%)	0.6419 ± 0.0179*** (+3.7%)
		Asian	0.5419 ± 0.0151	0.5434 ± 0.0163 (+0.2%)	0.5334 ± 0.0188 (−1.9%)	0.5267 ± 0.0171* (−3%)	0.5325 ± 0.0161 (−1.5%)

R0: Skin distensibility measured (mm). R2: Gross elasticity measured by Ua/Uf ratio. Data are means ± SEM. In brackets is reported the percentage variation versus baseline. Intragroup (vs. baseline) statistical analysis is reported as follows:

* *P* < 0.05,

**P < 0.01, and

*** *P* < 0.001.

ᶴ Statistically different versus placebo.

*Skin distensibility*, measured as R0 (mm), significantly decrease in the dietary supplement group (Table 3). This parameter is related to skin firmness, where decreased R0 indicates increased skin firmness. The overall (Caucasian and Asian subjects) skin distensibility decrease was by 3.8 (*P <* 0.0001), 4.7 (*P <* 0.0001), 6.7 (*P <* 0.0001) and 7.1% (*P <* 0.0001) after 2, 4, 8 and 12 weeks of treatment, respectively. Skin distensibility decrease in Caucasian subjects was by 3.0 (*P <* 0.0001), 4.1 (*P <* 0.0001), 6.1 (*P <* 0.0001) and 6.8% (*P <* 0.0001) after 2, 4, 8 and 12 weeks of treatment, respectively. Skin distensibility decrease in Asian subjects was by 4.5 (*P* = 0.0001), 5.3 (*P* = 0.0006), 7.2 (*P <* 0.0001) and 7.3% (*P* = 0.0005) after 2, 4, 8 and 12 weeks of treatment, respectively. No statistically significant differences were observed between Asian and Caucasian subjects in the active treatment group. However, in the placebo group a significant R0 decrease was observed in the Caucasian group, which was not observed in the Asian group. The skin distensibility variation observed in the active group was statistically significant when compared to the placebo treatment regimen, both overall (Caucasian and Asian subjects) and for Caucasian and Asian subjects at all checkpoints.

*The gross elasticity* (R2) parameter was significantly increased in the dietary supplement group (Table 3). The overall (Caucasian and Asian subjects) skin gross elasticity increase was by 2.9 (*P* = 0.0002), 6.1 (*P <* 0.0001), 7.1 (*P <* 0.0001) and 8.0% (*P <* 0.0001) after 2, 4, 8 and 12 weeks of treatment, respectively. Skin gross elasticity increase in Caucasian subjects was by 3.1 (*P <* 0.0001), 5.5 (*P <* 0.0001), 6.6 (*P <* 0.0001) and 7.6% (*P <* 0.0001) after 2, 4, 8 and 12 weeks of treatment, respectively. Skin gross elasticity increase in Asian subjects was by 2.6 (*P* = 0.1031), 6.8 (*P* = 0.0002), 7.7 (*P <* 0.0001) and 8.3% (*P <* 0.0001) after 2, 4, 8 and 12 weeks of treatment, respectively. The skin gross elasticity variation observed in the dietary supplement group was statistically significant when compared to the placebo treatment regimen, both overall (Caucasian and Asian subjects) at all the investigated checkpoints and for Caucasian and Asian subjects starting from 4 weeks of product use. No statistically significant differences were observed between Asian and Caucasian subjects in the active treatment group. However, in the placebo group, while in the Caucasian subgroup there was a significant increase in R0, this parameter was unchanged or even significantly reduced (8 weeks) among Asian subjects.

### Effects on radiance and skin colour

#### Skin brightness (gloss parameter)

**Fig. 4:**
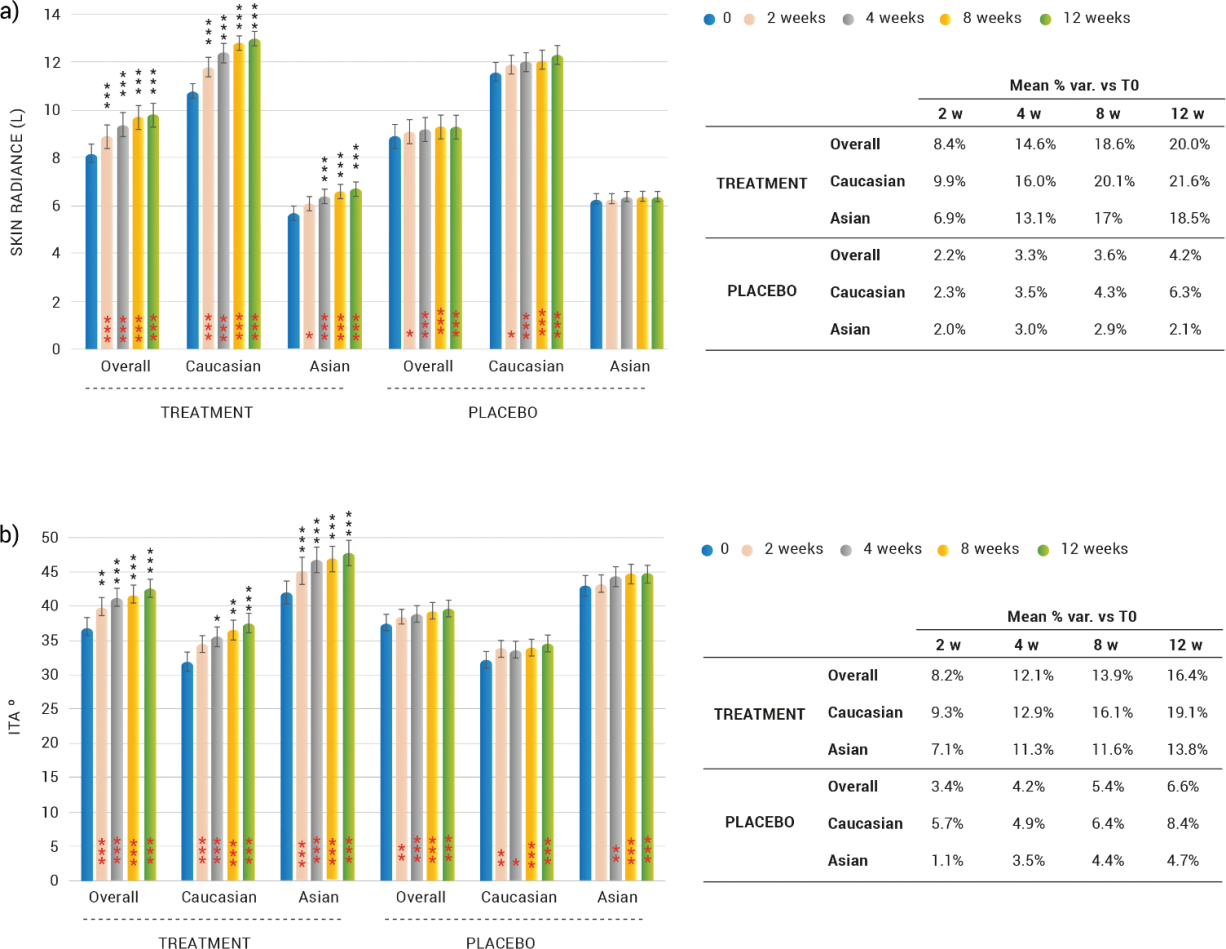
Effects on radiance and skin colour after 2, 4, 8, and 12 weeks of treatment. (a) Change on facial skin radiance. Data are means of gloss parameter (L) ± SEM in arbitrary units (a.u.). (b) Evolution of dark spot pigmentation. Data are presented as the means of ITA angle value ± SEM. Intragroup (vs. 0) statistical analysis is reported inside the bars of the histograms (in red). Intergroup (vs. placebo) statistical analysis is reported upon the bars of the histograms. Statistical analysis is reported as follows: *P < 0.05, **P < 0.01, and ***P < 0.001. In the tables are reported the percentage variation versus baseline.

Skin gloss, also referred to as skin radiance, was significantly increased both in Caucasian and Asian subjects (Fig. 4a). The overall (Caucasian and Asian subjects) skin radiance increase was by 8.4% (*P* < 0.0001), 14.6 (*P* < 0.0001), 18.6 (*P* < 0.0001) and 20.0% (*P* < 0.0001) after 2, 4, 8 and 12 weeks of treatment, respectively. Skin radiance increase in Caucasian subjects was by 9.9 (*P* < 0.0001), 16.0 (*P* < 0.0001), 20.1 (*P* < 0.0001) and 21.6% (*P* < 0.0001) after 2, 4, 8 and 12 weeks of treatment, respectively. Skin radiance increase in Asian subjects was by 6.9 (*P* = 0.0011), 13.1 (*P* < 0.0001), 17.0 (*P* < 0.0001) and 18.5% (*P* < 0.0001) after 2, 4, 8 and 12 weeks treatment, respectively. The skin radiance variation observed in the dietary supplement group was statistically significant when compared to the placebo treatment regimen, both overall (Caucasian and Asian subjects), in the Caucasian group at all the investigated checkpoints, and for Asian subjects after 4 weeks of product use. No significant differences were observed, in the radiance variation, between Asian and Caucasian subjects in the dietary supplement group. However, in the placebo group, while in the Caucasian subgroup there was a significant increase in skin radiance, this parameter was unchanged (*P* > 0.05) among Asian subjects.

#### Skin colour (ITA angle) on dark spot

A significant improvement (decrease in intensity of pigmentation) of dark spots, determined by ITA° measurement, was observed throughout the study both in Caucasian and Asian subjects (Fig. 4b). The overall (Caucasian and Asian subjects) melanin staining intensity decrease was by 8.2 (*P* = 0.0032), 12.1 (*P* < 0.0001), 13.9 (*P* < 0.0001) and 16.4% (*P* < 0.0001) after 2, 4, 8 and 12 weeks of treatment, respectively. Melanin staining intensity decrease in Caucasian subjects was by 9.3 (*P* = 0.0002), 12.9 (*P* < 0.0001), 16.1 (*P* < 0.0001) and 19.1% (*P* < 0.0001) after 2, 4, 8 and 12 weeks of treatment, respectively. Melanin staining intensity decrease in Asian subjects was by 7.1 (*P* < 0.0001), 11.3 (*P* < 0.0001), 11.6 (*P* < 0.0001) and 13.8% (*P* < 0.0001) after 2, 4, 8 and 12 weeks of treatment, respectively. In the placebo group, the mean variation in the percentage of the ITA measured in the brown spot presented a slight increase at the end of the study (6.6%), however, the melanin staining intensity variation observed in the dietary supplement group was statistically significant when compared to the placebo treatment regimen, both overall (Caucasian and Asian subjects) and for Asian subjects at all checkpoints, and for Caucasian subjects starting from 4 weeks of product use. No statistically significant differences were observed between Asian and Caucasian subjects in the ITA° variation.

### Effect on skin oiliness

A statistically significant decrease in the skin sebum content was observed both in Caucasian and Asian subjects (Table 4). The overall (Caucasian and Asian subjects) skin sebum content decrease was by 3.9 (*P* = 0.0010), 5.1 (*P* = 0.0001), 9.5 (*P* < 0.0001) and 10.9% (*P* < 0.0001) after 2, 4, 8 and 12 weeks of treatment, respectively. Skin sebum content decrease in Caucasian subjects was by 3.8 (*P* = 0.0175), 4.6 (*P* = 0.0056), 10.1 (*P* = 0.0002) and 12.0% (*P* = 0.0001) after 2, 4, 8 and 12 weeks of treatment, respectively. Skin sebum content decrease in Asian subjects was by 4.0 (*P* = 0.0101), 5.6 (*P* = 0.0030), 9.0 (*P* < 0.0001) and 9.8% (*P* = 0.0002) after 2, 4, 8 and 12 weeks of treatment, respectively. No statistically significant differences were observed between Asian and Caucasian subjects. The skin sebum content variation observed in the dietary supplement group was statistically significant when compared to the placebo treatment regimen, both overall (Caucasian and Asian subjects) starting from 8 weeks of product use and for Caucasian subjects after 12 weeks of product use.

**Table 4:** Effects on skin sebum content

Measurement	Groups		Intervention period				
			0 weeks	2 weeks	4 weeks	8 weeks	12 weeks
Sebum content (μg/cm^2^)	Treatment	Overall	127.4 ± 8.0	121.0 ± 6.8** (−3.9%)	118.6 ± 6.5*** (−5.1%)	113.1 ± 6.1*** (−9.5%)ᶴ	111.4 ± 5.9*** (−10.9%)ᶴ
		Caucasian	153.8 ± 13.4	145.5 ± 11.0* (−3.8%)	142.6 ± 10.4** (−4.6%)	135.1 ± 9.7*** (−10.1%)	132.4 ± 9.5*** (−12%)ᶴ
		Asian	100.9 ± 4.5	96.5 ± 4.3* (−4.0%)	94.6 ± 4.0** (−5.6%)	91.2 ± 3.9*** (−9.0%)	90.4 ± 4.1** (−9.8%)
	Placebo	Overall	142.5 ± 8.5	140.1 ± 8.5 (−1.3%)	137.2 ± 8.2*** (−3.3%)	133.4 ± 8.1*** (−5.9%)	132.4 ± 8.0*** (−6.4%)
		Caucasian	174.3 ± 14	171.2 ± 14.1 (−1.8%)	167.6 ± 13.2** (−3.9%)	162.8 ± 13.6*** (−6.8%)	160.9 ± 13.4*** (−7.8%)
		Asian	110.6 ± 4.1	109.0 ± 3.6* (−0.9%)	106.7 ± 3.5 (−2.7%)	104 ± 3.5* (−5.1%)	104.0 ± 3.5* (−5.0%)

Data are means of Sebum content (μg/cm^2^) ± SEM. In brackets is reported the percentage variation versus baseline. Intragroup (vs. baseline) statistical analysis is reported as follows:

* *P* < 0.05,

** *P* < 0.01, and

*** *P* < 0.001.

ᶴ Statistically different versus placebo.

### Subjective self-assessment of the properties of the product

The subjective and qualitative evaluation of the efficacy of the product showed that according to study subjects, the use of the dietary supplement was beneficial for their skin. The results of the questionnaire are shown in Table 5.

**Table 5:** Results of self-assessment questions for the treatment and placebo at week 4 and 12

Questions	Week 4		Week 12	
	Placebo	Treatment	Placebo	Treatment
My skin is protected from environmental pollution	58%	72%	62%	84%*
I perceive a protective sensation on my skin	64%	74%	64%	84%*
My complexion is more uniform	62%	74%	68%	84%
My skin is brighter	62%	74%	68%	76%
Wrinkles are less visible	62%	70%	60%	80%*
My skin is firmer	58%	70%	68%	82%
My skin complexion is healthier	62%	70%	64%	84%*
The colour of your skin is lighter (whiter skin)	52%	60%	52%	74%*
I feel my skin more moisturized	64%	76%	70%	86%*
I feel my hair healthier and stronger	54%	68%	62%	80%
I feel less discomfort related to pollutants-sensitivity	58%	70%	58%	82%*
*Reply among people with sensitive skin*	*60%*	80%*	*60%*	84%*
I notice an improvement of my skin imperfections (dilated pores and/or pimples)	58%	66%	68%	82%
I would buy the product	44%	56%	18%	82%*

The results of question 1 to 12 are expressed with the percentage of subjects who selected Strongly Agree or Agree to the questions presented. Question 13 are expressed as the percentage of subjects who would buy the product. Intergroup statistical analysis was performed between dietary supplement and placebo group (Caucasian+ Asian subjects) by means of Mann-Whitney statistical analysis.

(*) statistically significant *P* < 0.05.

All effects were more positively assessed by the treatment group as compared to the placebo group, largely at the end of the study with incremental improvements occurring from weeks 4 to 12. Of note, statistically significant differences between groups were found at the end of the study in 9 of the 13 questions. Regarding question 11, since 50% of the enrolled panel clinically presented sensitive skin, the answers were assessed by subdividing the subjects in sensitive/not sensitive skin. As a result, 80% of the subjects with sensitive skin perceived less discomfort relate to pollution-induced sensitivity only after 4 weeks. This difference was statistically significant compared with the placebo group both at week 4 and week 12.

## Discussion

The continual increase in air pollution worldwide is having a major negative impact on human health, including the skin. Although human skin shields against pollution, prolonged and repetitive exposure results in accelerated skin ageing, uneven skin pigmentation (8–11), inflammatory or allergic skin conditions such as atopic dermatitis and eczema, psoriasis, acne (12, 13) and even skin cancer (46). As a result, preventive and repair mechanisms to maintain optimal skin health and reverse the negative effects of air pollution, both topically and orally, are being given increased attention.

It is clear that air pollutants interfere with the normal functioning of the skin via oxidative damage to lipids, DNA and/or proteins (47–49). In addition, the effect of air pollutants can be amplified in the presence of UV radiation (15). Despite their different chemical nature and their mode of penetration into the skin, all air pollutants exert a harmful effect by increasing oxidative stress which counters the antioxidant defences of the affected tissue. There is a depletion of enzymatic (glutathione peroxidase, glutathione reductase, superoxide dismutase and catalase) and non-enzymatic (Vitamin E, Vitamin C and glutathione) antioxidant capacity (50, 51). Free radicals and ROS interact with the lipid-rich plasma membrane to initiate the LPO reaction cascade. Lipid peroxidation may contribute to and amplify cellular damage due to the generation of oxidized products, some of which are chemically reactive and covalently modify critical macromolecules (52). Reactive oxygen species (ROS) also stimulate the release of pro-inflammatory mediators which results in the accumulation of neutrophils and other phagocytic cells that further generate free radicals, thereby resulting in a vicious cycle. Thus, an anti-pollution ingredient’s effectiveness will depend on its antioxidant capabilities to counterbalance harmful substances.

Many studies suggest that the combination of natural products in appropriated formulations represent a viable strategy for the treatment of skin conditions associated with inflammation and oxidative stress, both topically and orally (19, 53). Published reports have shown that individual extracts or main constituents present in lemon verbena, olive leaf, *Sophora japonica* and rosemary are effective antioxidants with the ability to counteract different environmentally induced skin damage. The antioxidant effects of olive polyphenols, such as oleuropein and hydroxytyrosol, have been extensively confirmed in the scientific literature (29, 30). Several components in olive oil have a direct antioxidant effect on the skin, and its use has been suggested to prevent against chronic UV-induced skin damage, including melanin production (54–56). Hydroxytyrosol is also effective in reducing the harmful effect of light-mediated skin damage, including UVB (57), UVA (34) and blue light radiation (33). Studies have shown that rosemary extract exhibits strong antioxidant and anti-inflammatory properties linked to its polyphenol content, particularly diterpenes (carnosic acid and carnosol) (18, 38, 58, 59). Rosemary plant and carnosic acid have been proven to possess anti-photoaging properties and prevent the expression of matrix metalloproteinases (MMPs) in skin fibroblasts and keratinocytes (37, 60). Carnosic acid has also been shown to inhibit the oxidative stress levels on human fibroblasts exposed to UVA, urban dust and cigarette smoke (61). In other studies, the antioxidant and main biological properties of lemon verbena are linked to the presence of glycosylated phenylpropanoid, mainly verbascoside (62, 63). Verbascoside, also known as acteoside, has been proven to have a wide range of activities, including antioxidant, anti-inflammatory, photoprotective, whitening and chelating actions (35, 36, 64, 65). Finally, the role of *Sophora japonica* in Chinese Herbal Traditional Medicine is widely known, and numerous biological activities, such as antioxidant, anti-bacterial, anti-allergic, and anti-inflammatory, have been reported (31). In addition, the photoprotective and anti-pollution effects of *Sophora japonica* have also been studied (32, 66).

Skin exposure to PMs and other pollutants has been found to induce LPO, as well as elevated levels of reactive aldehyde by-products such as malondialdehyde (MDA) (4). On the other hand, total antioxidant capacity (TAC) is a recognized biomarker for measuring the antioxidant potential of body fluids that is also used to investigate oxidative stress in many pathological conditions (67). Environmental pollutants can also affect TAC. Smoking, O_3_, heavy metals and toxic elements such as pesticides can decrease TAC, rendering subjects less resistant to oxidative and nitrosamine injuries and subsequent diseases (68–71). The results of the present study showed that the oral intake of the product containing the four herbal extracts ingredient improved oxidative stress biomarkers by significantly reducing the MDA content on the skin stratum corneum and increasing the TAS after 12 weeks and 4 weeks of product consumption, respectively. The increase of the TAS and the decrease of the skin LPOs indicate a positive effect of the product in improving the body’s antioxidant capacity and interestingly, its targeting to the skin. The decrease of the oxidative stress in the skin could be a first step in decreasing the generation of free radicals by PM, O_3_ and other oxidants in the air pollution. On the other hand, the replenishment of antioxidants by dietary ingredients could be of primary importance in decreasing the ROS-mediated depletion and both enzymatic and non-enzymatic antioxidant reserves in the skin with notable effects on Vitamin C and E levels (51, 72).

The damaged barrier leads to higher TEWL, resulting in poor skin hydration causing dry and sensitive skin. Strengthening the skin’s barrier and improving hydration is also a promising antipollution strategy (42, 43). Pollution weakens the skin’s natural barrier, resulting in higher and deeper penetration and accumulation of pollutants, which, in turn, affects the morphology and integrity of skin structure (73). In this study, a positive effect of the product was also seen on skin parameters related to the skin’s barrier integrity where the improvement of skin moisturization correlated with TEWL decrease. A positive correlation was also seen in the itching sensation of people with sensitive skin as we showed in the subject self-assessment questionnaire.

Pollutant exposure has been linked with premature skin ageing. In the present study, an improvement of the skin’s firmness and elasticity were observed. Additionally, the skin became smoother and the depth of the wrinkles was reduced in the crowfeet area. These improvements were observed after only 2 weeks of taking the dietary supplement.

Although the underlying mechanisms by which the active ingredient improves skin wrinkles, hydration, elasticity, and roughness were not investigated in this clinical trial, we suggest that the polyphenol-enriched dietary supplement may have a positive effect on the extracellular matrix status through the antioxidative activities of the phenolic compounds present in the active formulation. Particulate matters and other pollutants induce ROS and inflammatory mediators that produce the activation of nuclear transcription complex AP-1, through intracellular kinases signalling activation (MAP kinases, p38 and JNK), leading to MMPs activation and decreased expression of collagen and other matrix proteins with the final consequence of reduced dermal matrix formation (74).

A positive correlation was also observed for the improvement of overall skin complexion. The daily plant-based nutraceutical consumption provided exceptional skin brightening benefits (five times more than the placebo group) and significantly lightened the hyperpigmented areas on the cheekbone (2.5 times more than the placebo group). These improvements were statistically significant compared to the placebo group after only 2 weeks of taking the dietary supplement.

In addition to the antioxidant effect, the beneficial properties of the polyphenolic blend may be mediated through the inhibition of the AhR activation induced by pollutants. The aryl hydrocarbon receptor is a ligand-activated transcription factor found in various skin cells, including keratinocytes, fibroblasts, melanocytes and Langerhans cells. Non-activated AhR is trapped in the cytosol but under the effect of various environmental aggressors (i.e. UV radiation, O_3_, tobacco smoke and air pollutants, especially PAH-rich PM) AhR translocates to the nucleus where it stimulates the expression of genes containing the xenobiotic response. Some of these genes control the expression of proteins involved in oxidative stress reactions, inflammation, immunosuppression, pigmentation, premature ageing and cancer in the skin (16, 75–77). Different studies have proven the capacity of different polyphenols present in the formula to inhibit the AhR activation in response to toxic substances and UV radiation. That is the case of quercetin (78–80), verbascoside (79) and carnosol (81, 82). Also in an unpublished study, the polyphenol-enriched dietary supplement was shown to decrease significantly the AhR expression in human skin explants exposed to pollutants. However, more research is needed to prove this hypothesis.

In this clinical trial, the benefits of the product were substantiated by a self-assessment questionnaire as the treatment product was highly rated by volunteers regarding its efficacy. Of note, 82% of participants in the treatment group would purchase the product. The importance of the user opinion, especially having a nutraceutical ingredient with cosmetic properties cannot be underestimated because it helps to comply with the product adherence.

The strengths of this study include the randomized, double-blind, placebo-controlled design and use of an equal distribution of women across a broad range of age groups, skin type, skin sensitivity. In addition, compared to other studies performed in this area, the study population size was relatively large and included both Asian and Caucasian subjects. Additional strengths include the control of external variables (e.g. sun or tanning bed exposure, dietary supplement use, etc.) that could interfere with the observed results, as well as utilization of standardized facial cleanser and moisturizer throughout the study. Despite these strengths, some limitations that are worth noting, for instance, the regulatory authorities have not yet established standardized methods for validating the antipollution efficacy of cosmetic or nutritional products on human skin. Furthermore, all the volunteers in the clinical trial were female, however, we believe that our results can be extended to the general population since the molecular, cellular, and tissue-specific events leading oxidation, inflammation, and skin ageing are shared among genders.

The main objective of this study was to evaluate the ability of the multi-herbal active ingredient to protect the skin of subjects living in a highly polluted environment during the winter season, a time frame characterized by high air pollution (especially PM10 and PM2.5). During the study evaluation, high pollution days (exceeding the WHO guideline) represented 90 and 96% of the entire study period for PM 10 and PM 2.5, respectively. This supports the fact that we tested the skin protective effect of the dietary supplement under a suitable time frame to evaluate its antipollution effect. To our knowledge, this is the first clinical study set out to explore the efficacy of a nutraceutical ingredient to prevent the harmful effects of air pollutants on the skin.

## Conclusion

The results of the study indicate a positive effect of the dietary supplement in improving the skin conditions of both Asian and Caucasian females living in a polluted urban area.

After 12 weeks of product use, the polyphenol-enriched dietary supplement provided significant improvements compared to the placebo in all clinically measureable efficacy parameters including skin lipoperoxides. Positive effects such as decreased wrinkle depth, increased elasticity and firmness, strengthened skin barrier function and reduced dark spots were noted as short as 2 weeks of product consumption.

In conclusion, the long-term oral intake of Zeropollution^®^ could be considered a complementary nutrition strategy to protect the skin cells from pollution-induced oxidative stress and skin ageing.
